# What About the Differences in Body Representation and Body Image Between Stroke Patients and Healthy Controls? Psychological and Clinical Implications

**DOI:** 10.1002/brb3.70155

**Published:** 2025-05-08

**Authors:** Maria Grazia Maggio, Amelia Rizzo, Morena De Francesco, Martina Barbera, Muhammad Kamran, Rosaria De Luca, Francesco Corallo, Angelo Quartarone, Rocco Salvatore Calabrò

**Affiliations:** ^1^ IRCCS Centro Neurolesi Bonino Pulejo Messina Italy; ^2^ Department of Clinical and Experimental Medicine University of Messina Messina Italy; ^3^ Department of Education University of Loralai Loralai Balochistan Pakistan

**Keywords:** body image, body representation, neurological patients, neurorehabilitation, stroke

## Abstract

**Introduction:**

The integrity of body representation can be profoundly compromised in neurological patients. This study aims to evaluate body representation in both healthy individuals and stroke patients.

**Material and Methods:**

The study included 40 chronic stroke patients recruited from the Neurorehabilitation Unit, IRCCS Centro Neurolesi “Bonino‐Pulejo,” and 40 healthy controls from the University of Messina. Both groups were age‐ and gender‐matched. All participants completed the Body Uneasiness Test to assess body image discomfort and body schema accuracy.

**Results:**

A total of 80 participants were enrolled, divided equally into two groups matched by gender: stroke patients and healthy controls. Healthy controls showed higher scores in compulsive self‐monitoring (*p *= 0.023), while stroke patients exhibited greater depersonalization (*p *= 0.039) and significantly higher psychological distress (*p* ≤ 0.001).

**Discussion:**

These findings underscore the complexity of bodily and psychological experiences in both health and disease, emphasizing the need for tailored clinical interventions. While the direct impact on quality of life may vary, addressing these issues can contribute to improved overall well‐being for both groups.

## Graphical abstract

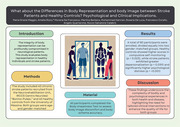

## 1 Introduction

Body representation and body image are fundamental concepts in the study of psychology and neuropsychology, as they profoundly influence how individuals perceive themselves and interact with the world (Schwoebel and Coslett 2005). Body representation refers to the internal processing of how the body is perceived and represented in the brain, whereas body image focuses on the conscious perceptions, attitudes, and thoughts that an individual has about their body (Giummarra et al. 2008; Schwoebel and Coslett 2005). These concepts are not static but can be significantly altered by various factors, including neurological experiences, such as stroke (Fang, Liu, and Wang 2024; Maggio, Naro, Calatozzo, et al. 2021).

In neurological patients, and particularly those affected by stroke, the integrity of the body representation can be profoundly compromised (Maggio, Naro, Manuli, et al. 2021; Razmus 2017). Stroke, which affects brain areas critical for sensorimotor processing and body awareness, can lead to disorders such as anosognosia, in which the patient is unaware of their deficits, or unilateral spatial neglect, in which a body part is ignored (Maggio, Naro, Manuli, et al. 2021; Razmus 2017). These disorders not only affect functional capacity but also body perception, contributing to distortions in body image and a fragmented body identity (Maggio, Naro, Manuli, et al. 2021; Maggio, Naro, Calatozzo, et al. 2021).

The literature indicates that in patients with stroke and other neurological conditions, body image can undergo negative transformations, characterized by an altered perception of affected body parts, which may be experienced as foreign or not belonging to the self, or may be experienced negatively, with hatred or disdain (Maggio, Naro, Calatozzo, et al. 2021). This can lead to serious psychological implications, including anxiety, depression, and a decreased quality of life. Furthermore, the poststroke recovery process, which often requires relearning motor skills and reconceptualizing one's body, can further complicate the perception of body image, making rehabilitation not only a physical but also a psychological challenge (Maggio et al. 2022; Thomas and Lincoln 2006).

On the other hand, even in healthy subjects, body representation and body image can be influenced by psychological and cultural factors, such as social pressure and the media, which often promote unrealistic body ideals (Grogan 2021). These factors can induce excessive concerns about weight, body shape, and physical appearance, contributing to dysfunctional behaviors such as compulsive body monitoring and social avoidance (Grabe, Ward, and Hyde 2008). Such concerns are not simply a reflection of vanity, but represent a significant dimension of mental health, with potential impacts on life satisfaction and general well‐being (Bandura 1997; Grogan 2021).

In this context, it is crucial to understand how body representation and body image differ between healthy individuals and those with neurological conditions, such as stroke. Exploring these differences not only enriches our understanding of the psychological implications of these conditions but can also guide the development of clinical interventions to improve the quality of life of both neurological and healthy patients. Therefore, our study aims to evaluate aspects concerning body representation in healthy individuals and stroke patients.

## 2 Materials and Methods

This study is an observational comparative study, conducted in Italy, designed to evaluate psychological distress and body image concerns among individuals with chronic stroke. A cross‐sectional research design was employed, allowing for a simultaneous assessment of the two participant groups at a specific point in time. The study involved two groups of participants. Group 1 (ST‐G) comprised 40 individuals affected by chronic stroke, recruited first from the Neurorehabilitation Unit, IRCCS Centro Neurolesi “Bonino‐Pulejo,” between January 2020 and March 2023. Group 2 (HC‐G) consisted of 40 healthy controls, matched for age and gender, recruited subsequently from the University of Messina during the same timeframe. Both groups were matched for age and gender to ensure comparability. The sample size for groups was determined based on a lower variability in body representation studies, necessitating 40 participants to achieve a significant effect size with an 80% power and a significance level of 0.05. Consequently, we aimed to recruit 40 participants for each group for our study to ensure the adequacy and reliability of our findings. After recruitment, participants completed the Body Uneasiness Test (BUT) (Cuzzolaro et al. 2006), which was administered in a standardized manner by a skilled psychologist to ensure consistency.

For stroke participants, the inclusion criteria were as follows: (i) age between 18 and 80 years; (ii) unilateral hemiparesis resulting from a first‐ever supratentorial stroke occurring within 6 months before enrolment (i.e., in the subacute phase); and (iii) ability to follow verbal instructions, with a Montreal Cognitive Assessment (MoCA) score greater than 24.

Exclusion criteria were as follows: (i) history of multiple strokes or strokes not fitting the criteria of first‐ever supratentorial stroke; (ii) MoCA score of 24 or below, indicating impaired cognitive function; (iii) presence of significant comorbidities or medical conditions that could impact participation or performance in the study; and (iv) difficulty in following verbal instructions that could affect participation in the study.

For healthy participants, the inclusion criteria were: (i) age between 18 and 80 years; (ii) no history of neurological disorders, including stroke, traumatic brain injury, or neurodegenerative diseases; (iii) good general health with no significant medical conditions that could affect body representation or performance on the BUT; (iv) no other cognitive impairments or psychiatric conditions that could influence responses on the BUT; (v) ability to provide informed consent, understanding the study's purpose and procedures; and (vi) sufficient proficiency in Italian to understand the test instructions and questions.

Exclusion criteria were as follows: (i) history of neurological disorders, including stroke, traumatic brain injury, or neurodegenerative diseases; (ii) significant medical conditions or severe comorbidities that could impact participation or performance in the study; and (iii) cognitive impairments or psychiatric conditions affecting the ability to understand or respond to the BUT.

### 2.1 Outcome Measures

After recruitment, participants completed the BUT, which was administered in a standardized manner by a skilled psychologist to ensure consistency across all assessments. The primary outcome measure for this study was BUT, which evaluates various dimensions of body representation. The BUT is divided into two main components.

The first component, BUT‐A: Body uneasiness, focuses on measuring the level of discomfort and distress related to body image. It evaluates emotional and psychological unease concerning one's body, including perceptions of body distortion and dissatisfaction with body appearance. Participants respond to statements reflecting their feelings about their body image, capturing any anxiety or discomfort experienced regarding their physical appearance and related thoughts.

The second component, BUT‐B: Body schema, examines the accuracy and coherence of the individuals' body schema. It assesses how well individuals can perceive and mentally represent their body's shape, size, and spatial orientation. Participants answer questions about their perceptions of body size and shape discrepancies, as well as their spatial awareness of their body. This component helps identify any distortions in body schema that may arise from neurological conditions.

The responses were scored to determine levels of body uneasiness and body schema distortions. Higher scores on the body uneasiness subscale indicate greater discomfort with body image, while discrepancies in the body schema subscale reveal inaccuracies in body perception.

### 2.2 Statistical Analysis

Data were analyzed using SPSS 27.0 (IBM). The normality of the data distribution in the ST‐G and HC‐G was assessed via skewness and kurtosis values. For the ST‐G group, significant non‐normality was observed in most variables, with skewness values, such as 2.47 for “BUT‐A Avoidance,” 1.90 for “BUT‐B II Face Shape,” and 1.56 for “BUT‐B III Thighs,” indicating substantial asymmetry (values > ±1). Corresponding kurtosis values, such as 8.74 for “BUT‐A Avoidance,” and 3.85 for “BUT‐B II Face Shape,” further suggested leptokurtic distributions with pronounced peaks (values > ±3).

HC‐G also exhibited significant skewness and kurtosis for several variables, reinforcing the presence of non‐normal distributions, further indicating non‐normal distributions. Given these findings, non‐parametric statistical methods were applied to ensure appropriate analysis. Descriptive statistics were presented as mean ± standard deviations, and categorical variables were reported as frequencies and percentages. Differences between the two groups were assessed using the Mann–Whitney *U* test. Statistical significance was adjusted to *p* < 0.03 following Bonferroni correction.

## 3 Results

A total of 80 participants were enrolled in the study, divided equally into two groups of 40 each. The gender distribution between the two groups was comparable, with an identical ratio of females to males. This ensured that gender did not confound the outcomes of the comparative analyses.

Regarding age, both groups display comparable distributions, with only slight variations in the mean and maximum ages, which are not statistically significant. The analysis confirms that the demographic characteristics of the participants are well‐matched, minimizing potential biases in the study results. Further details can be found in Table 1.

**TABLE 1:** Demographic characteristics of the sample.

	ST‐G	HC‐G	All	*p*‐value
Subjects	40	40	80	—
Age (years)	52.9 ± 15.2	51.9 ± 14.4	52.4 ± 14.9	0.64
Male (%)	23 (57.5%)	23 (57.5%)	46 (57.5%)	1.00
Education (years)	17.2 ± 2.8	17.3 ± 3.4	17.1 ± 3.1	0.70
Interval from stroke				
Mean in months	6 ± 1	—	—	—
Brain lesion site/side				
Cortical right	24 (60%)	—	—	—
Subcortical right	12 (30%)	—	—	—
Cortical left	4 (10%)	—	—	—
Subcortical left	0	—	—	—

*Note*: Mean ± standard deviation was used to describe continuous variables (age and education); proportions (numbers and percentages) were used to describe categorical variables (gender and brain lesion). ST‐G (Group 1: stroke‐group); HC‐G (Group 2: health control‐group).

The results of independent‐samples Mann–Whitney *U* test showed statistically significant differences between ST‐G and HC‐G across various psychological and symptomatic indices (Table 2).

**TABLE 2:** Descriptive of BUT‐A dimensions across groups.

	ST‐G		HC‐G	
	Av	SD	Av	SD
BUT•A Weight phobia	0.96	0.80	0.34	0.98
BUT •A Body image concern	0.99	0.80	0.28	0.92
BUT •A Avoidance	0.78	0.96	0.64	0.77
BUT •A Compulsive self‐monitoring^a^	0.65	0.43	0.02	0.69
BUT •A Depersonalization^a^	0.82	0.68	0.60	0.85
GSI^a^	0.88	0.60	0.04	0.77
PST	0.70	0.62	0.75	0.55
PSDI^b^	0.48	0.31	0.35	0.16

Abbreviations: AV, average; BUT, Body Uneasiness Test; GSI, Global Severity Index; HC‐G, healthy controls group; PSDI, Positive Symptom Distress Index; PST, positive symptom total; SD, standard deviations; ST‐G, stroke patients group.

^a^ Significance < 0.03.

^b^ Significance < 0.001.

**FIGURE 1:**
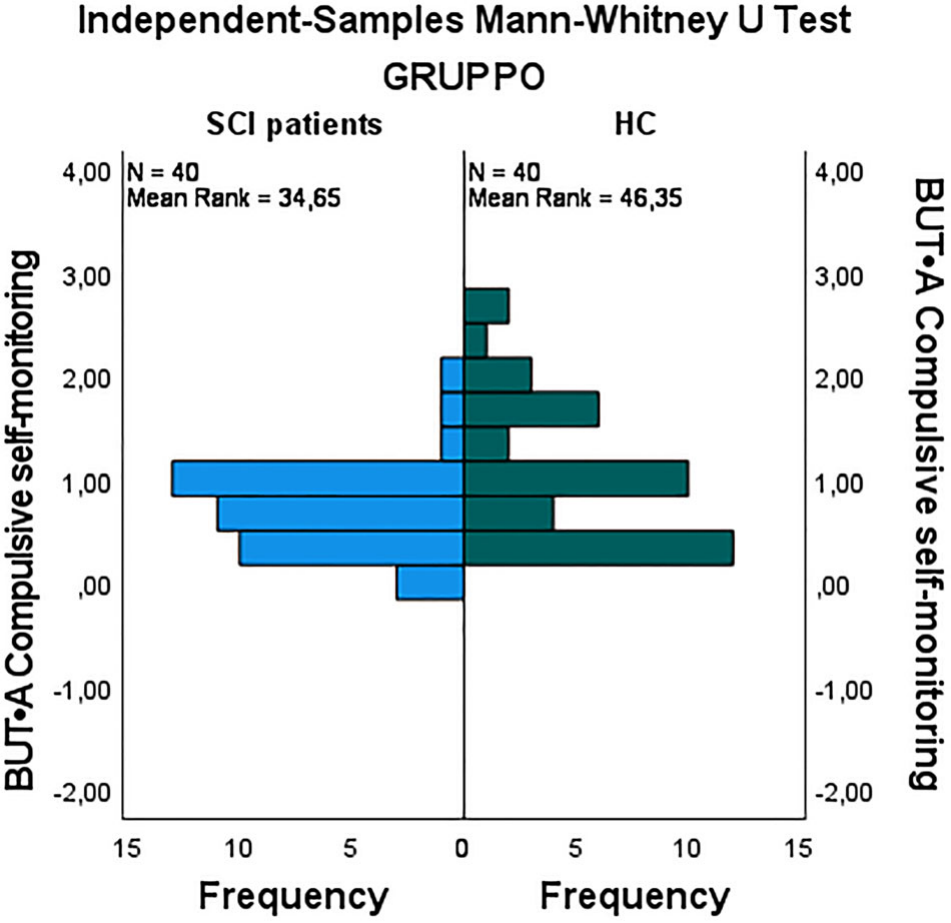
Comparison of compulsive self‐monitoring between stroke group (ST‐G) and healthy control group (HC‐G).

**FIGURE 2:**
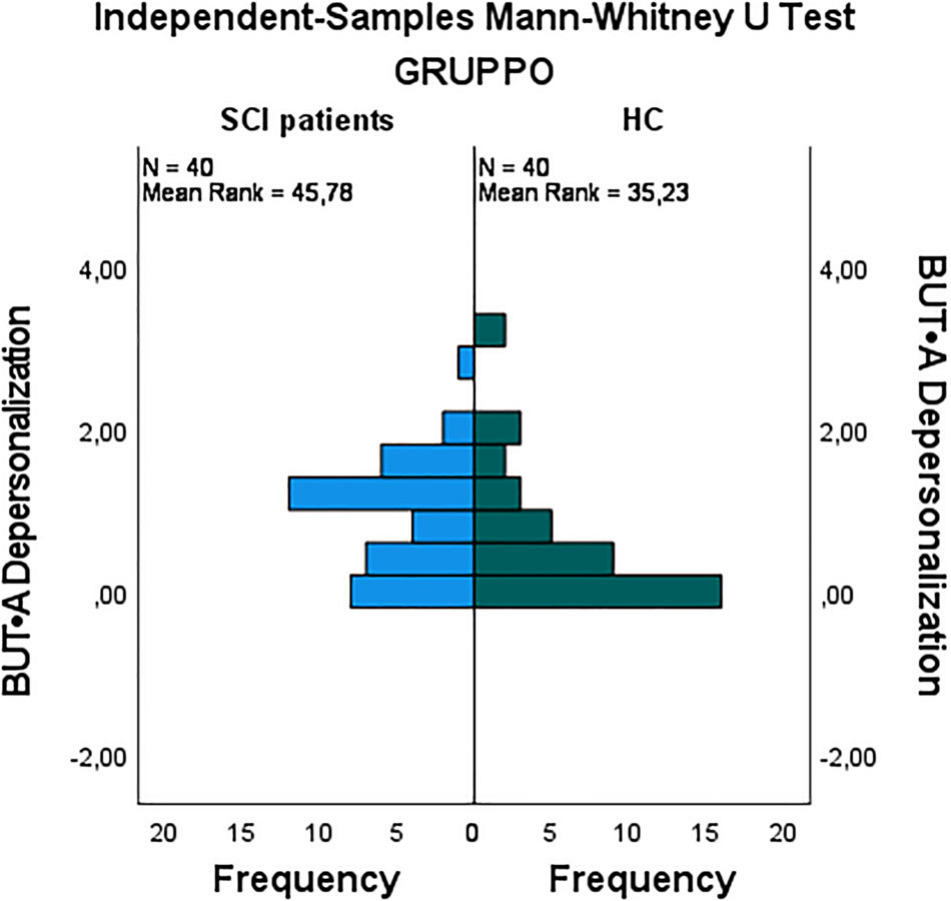
Comparison of depersonalization between stroke group (ST‐G) and healthy control group (HC‐G).

**FIGURE 3:**
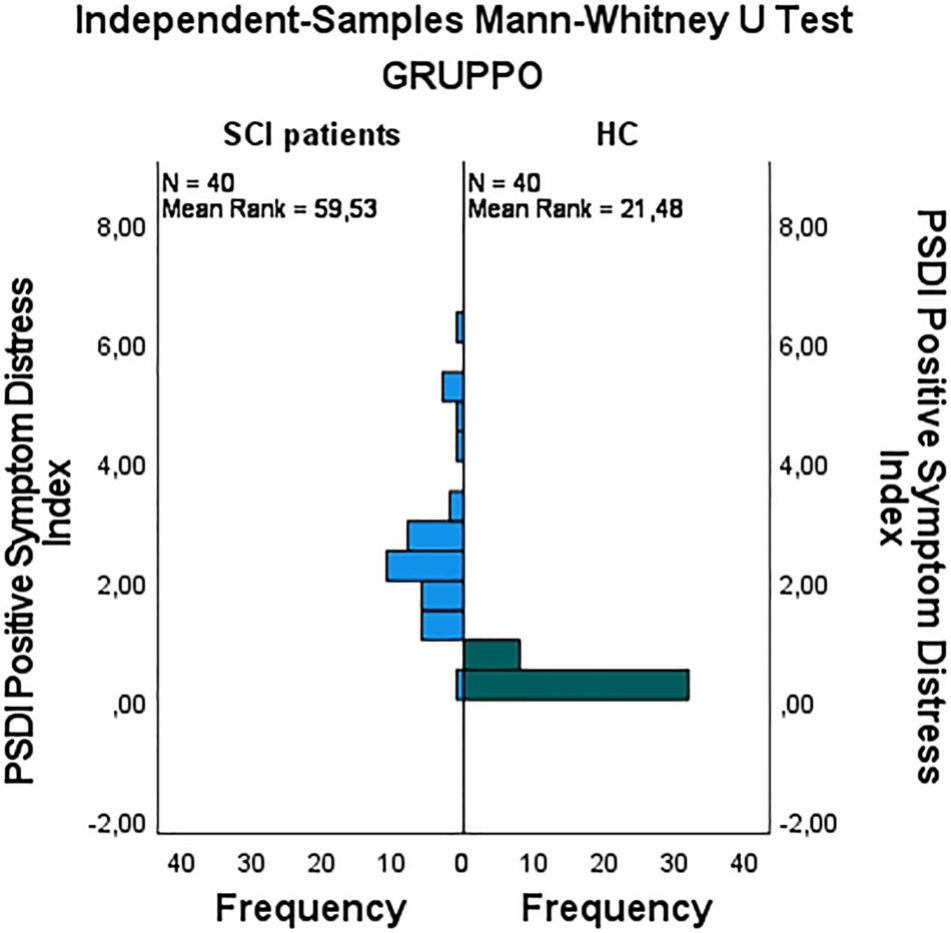
Comparison of positive symptom distress index between stroke group (ST‐G) and healthy control group (HC‐G).

HC‐G exhibited higher mean scores in compulsive self‐monitoring (1.02 vs. 0.65), with this difference reaching statistical significance (*p *= 0.023) (Figure 1). Additionally, ST‐G demonstrated higher scores in depersonalization (0.82 vs. 0.60), which was statistically significant (*p *= 0.031) (Figure 2). Finally, the Positive Symptom Distress Index (PSDI) was significantly elevated in stroke patients (2.48 vs. 0.35, *p* < 0.001) (Figure 3). These findings underscore that stroke patients experience greater psychological distress in specific domains compared to healthy controls (Figure 3).

In addition, the discomfort related to the legs among ST patients (mean = 1.04) and blushing (mean = 1.16) were reported as clinically significant, though these differences did not reach statistical significance. The discomfort in the leg mobility is associated with stroke, highlighting the presence of motor deficits. Notably, discomfort associated with blushing could suggest increased emotional awareness or social sensitivity, potentially due to altered body perception following stroke.

When examining the discrepancies in discomfort levels between ST‐G and HC‐G, it is clear that while not statistically significant, these differences carry substantial clinical relevance. Among the healthy control group, concerns primarily centered around skin discomfort (mean = 1.04). However, for the ST patients, significant discomfort in the legs (mean = 1.04) and blushing (mean = 1.16) were particularly notable (see Table 3).

**TABLE 3:** Descriptive of BUT‐B dimensions across groups.

	ST‐G		HC‐G	
	Av	SD	Av	SD
BUT ● B I Mouth	0.58	0.51	0.61	0.64
BUT ● B II Face shape	0.19	0.28	0.39	0.49
BUT ● B III Thighs	0.74	0.85	0.96	0.90
BUT ● B IV Legs	1.04	0.91	0.85	0.84
BUT ● B V Harms	0.32	0.46	0.67	0.88
BUT ● B VI Mustache	0.49	0.70	0.33	0.53
BUT ● B VII Skin	0.78	0.88	1.04	1.32
BUT ● B VII Blushing	1.16	0.83	0.89	1.01

Abbreviations: Av, average; BUT, Body Uneasiness Test; HC‐G, healthy controls group; SD, standard deviations; ST‐G, stroke patients group.

Significance < 0.03.

Significance < 0.001.

The discomfort in the legs may suggest mobility impairments linked to motor deficits in stroke patients. However, the most intriguing finding concerns the reported discomfort related to blushing in ST patients, which may indicate heightened emotional sensitivity or altered body perception.

## 4 Discussion

Our findings provided new insights into body image and psychological distress in patients with stroke and healthy subjects. In particular, the differences observed between the two groups in terms of body image concerns, avoidance behaviors, and depersonalization symptoms highlight how the brain injury affects body perception and psychological well‐being in distinct ways.

Our results could reflect an altered or heightened body perception following stroke, affecting physical functionality, self‐perception, and body image. For example, discomfort related to the legs might stem from the loss of mobility or a sense of detachment from body parts that are no longer under voluntary control. Similarly, the higher score in “blushing” could indicate increased emotional awareness or social sensitivity in situations that evoke embarrassment or stress. This result could suggest that the brain injury could alter body perception and self‐image, potentially leading to heightened psychological distress.

This is consistent with studies demonstrating that physical injury often leads to heightened awareness of bodily sensations and emotional responses (Fang, Liu, and Wang 2024; Maggio, Naro, Calatozzo, et al. 2021). Brooks et al. (2016) noticed that individuals with neurological impairments often experience heightened sensitivity to physical and emotional stimuli, which can exacerbate feelings of distress. In addition, Oouchida et al. (2016) showed that brain injuries can lead to altered self‐perception and body image, contributing to increased psychological challenges. Indeed, in our sample, discomfort related to the legs might stem from the loss of mobility or a sense of detachment from the body parts that are no longer under voluntary control. This aligns with findings from Gallagher and Cole (1995) who demonstrated that the loss of motor function can result in altered sensory and perceptual experiences of body parts.

Similarly, in our sample, we observed that higher scores in “blushing” could indicate increased emotional awareness or social sensitivity. This finding raises pertinent questions about the affective domain. Blushing is, indeed, one of the physical manifestations of the emotion of shame. This suggests that stroke can profoundly disturb, extending beyond mere physical disability as it impacts the emotional sphere. This finding might be linked to the research by Rosen and Levenson (2009), who found that neurological conditions often amplify emotional responses and social sensitivity. Moreover, studies by Morin (2017) highlights that neurological impairments frequently lead to altered body image and heightened psychological stress, reflecting the complex interplay between physical and psychological aspects of body perception. Furthermore, Obrero‐Gaitán et al. (2024) and Saeys et al. (2012) emphasize that individuals with neurological injuries often experience intensified distress due to changes in body functionality and self‐perception. Indeed, our patients with stroke reported higher scores in avoidance behaviors, depersonalization, distress associated with positive symptoms, and discomfort related to some parts of their body. Particularly, the PSDI showed the most significant statistical difference between stroke patients and healthy controls, emphasizing the substantial psychological burden experienced by individuals with stroke. This index reflects the intensity of psychological symptoms and provides insight into the overall emotional distress faced by stroke survivors. The marked elevation of PSDI in the stroke group underscores the need to address not only the physical but also the emotional consequences of stroke. This finding aligns with our initial hypothesis that psychological distress would be heightened in the stroke population due to the profound changes in body perception and functionality. The strong association between elevated PSDI scores and stroke‐related body image disturbances suggests that rehabilitation programs should integrate psychological support focused on alleviating emotional distress, which may improve overall recovery outcomes.

These findings suggest that brain injury may lead to a disconnection and depersonalization from one's body, likely due to the physical limitations and functional changes imposed by the medical condition. Depersonalization is a well‐known phenomenon in the psychopathology associated with neurological conditions (Sattin et al. 2023; Sierra and Berrios 2002). Depersonalization can be interpreted as a defense mechanism that protects the individual from confrontation with a body that no longer responds as it once did (Ciaunica et al. 2024). Studies by Sierra and Berrios (2002) have demonstrated that patients with brain injury or significant trauma often develop symptoms of depersonalization as part of a dissociative response to trauma, which aligns with our findings. The research underscores that depersonalization may help individuals manage the psychological impact of altered body functionality and trauma (Sattin et al. 2023). Indeed, existing literature supports our observations regarding differences in body perception between stroke patients and healthy subjects. Several studies have shown that brain injuries, including stroke, often lead to a disconnection between the body and the mind, a phenomenon that has been linked to depersonalization and an emotional detachment from the body (Jehkonen, Laihosalo, and Kettunen 2006; Llorens et al. 2017; Maggio, Naro, Calatozzo, et al. 2021; Morin 2017; Saeys et al. 2012). Jehkonen, Laihosalo, and Kettunen (2006) documented that stroke patients tend to develop disturbances in body perception, including a distorted perception of the size and shape of their body, like what we found in our study. Furthermore, the avoidance behavior observed in stroke patients, as documented in our research, finds support in the literature. Previous studies have suggested that avoidance may be a coping strategy used by patients to reduce the distress associated with the perception of a body no longer functioning as before. Indeed, social avoidance and avoidance of situations that require body awareness have been documented in patients with paralysis or other severe physical disabilities (Stott et al. 2021; Timothy, Graham, and Levack 2016).

Notably, in our study, the distribution of brain lesions among stroke patients reveals significant information about how different areas of brain injury might affect body representation and psychological well‐being. Patients with right cortical lesions (24 patients) are more likely to experience disruptions in spatial awareness and body schema. The right cortex plays a crucial role in the integration of sensory information and spatial processing, and lesions in this area can lead to impaired body perception and increased psychological distress (Fiorilli et al. 2021). The involvement of the right subcortical regions (12 patients), such as the basal ganglia and thalamus, adds another layer of complexity. These structures are involved in motor control and sensory integration, and their impairment may indirectly affect body awareness and emotional regulation (Köhler et al. 1995).

In contrast, left cortical lesions (four patients) may primarily affect fine motor control and language, with a less direct impact on body representation than right hemisphere lesions (Abdoli et al. 2024). Although the number of patients with left subcortical lesions is zero in our study, it is known that lesions in subcortical structures can still affect motor function and emotional regulation, potentially influencing body representation in complex ways (Fiorilli et al. 2021; Hamamoto et al. 2023). Overall, these findings suggest that right hemisphere lesions, particularly in cortical and subcortical regions, may be associated with disruptions in body representation and emotional processing. However, the impacts on body representation are rarely considered during patient rehabilitation. This highlights the importance of considering the location of the lesion and its impact on body image and psychological well‐being when designing rehabilitation programs for stroke patients.

Finally, a key finding of our study is the significant difference between the two groups in terms of body image concerns. Both groups show difficulties and concerns regarding body image, but the nature of these concerns varies significantly. For stroke patients, body image concerns are often related to depersonalization, embarrassment, or avoidance of certain body parts, which can be attributed to changes following the neurological injury. In contrast, healthy individuals mainly experience concerns related to body image itself, such as weight phobia and dissatisfaction with appearance. This distinction highlights how neurological injuries can lead to unique psychological challenges compared to those faced by healthy individuals, who are more influenced by social standards and personal body image ideals. Indeed, healthy subjects showed higher scores in weight phobia, body image concerns, and compulsive self‐monitoring than neurological patients. This finding suggests that healthy individuals may experience greater body awareness and sensitivity to social norms about body appearance, as they do not have to deal with the physical challenges of a neurological condition. Some studies supported this hypothesis, indicating that social and cultural pressures can have a significant impact on body dissatisfaction, particularly in Western populations (Abdoli et al. 2024; Neumark‐Sztainer et al. 2006). Grogan (2006) highlighted that weight phobia and body image concerns are prevalent among individuals influenced by media‐promoted beauty ideals. This aligns with our observation that healthy subjects have greater body image concerns than Stroke patients. Further studies, such as those by Groesz, Levine, and Murnen (2002) have shown that media exposure contributes to body dissatisfaction and increased attention to appearance concerns. Similarly, Stice et al. (2015) highlighted that the internalization of society's beauty standards is associated with increased body dissatisfaction and disordered eating behaviors. Furthermore, Perloff (2014) noticed that social comparison and pressure to conform to esthetic ideals are significant predictors of body image problems.

### 4.1 Clinical Application

The differences observed between the ST‐G and HC‐G groups support the need for tailored therapeutic interventions that address specific body image concerns and psychological impacts associated with each group.

For stroke patients, the focus should be on enhancing body awareness and addressing symptoms of depersonalization. This could involve interventions that improve body perception and reduce avoidance behaviors. Techniques such as robotics and virtual reality rehabilitation have shown promise in helping Stroke patients regain a sense of body ownership and reduce psychological distress (Maggio,, Naro, Manuli, et al. 2021; Maggio, Naro, Calatozzo, et al. 2021; Maggio et al. 2022). These methods may assist in bridging the gap between the altered body experience and psychological well‐being.

For healthy individuals, it is crucial to proactively address body image concerns to prevent potential issues such as eating disorders or low self‐esteem. Implementing health education programs that promote a positive body image and challenge unrealistic aesthetic ideals can be beneficial. Such programs can play a preventive role in mitigating body image issues before they escalate into more serious psychological disturbances (Yager and O'Dea 2010).

By addressing these issues through targeted interventions and preventative measures, both stroke patients and healthy individuals can benefit from improved psychological well‐being and body image.

### 4.2 Strengths and Limitations of the Study

This study offers valuable insights into the psychological distress and body image concerns in stroke patients, utilizing a robust comparative methodology. A notable strength is the inclusion of age‐ and sex‐matched healthy participants, which enhances the validity of the findings. This design allows for a clearer contextual understanding of the unique challenges faced by individuals with neurological impairments, setting this study apart from traditional cross‐sectional research. By contrasting the experiences of stroke patients with healthy controls, the study provides a more nuanced examination of the impact of stroke on body perception and emotional well‐being. These findings highlight the importance of addressing both physical functionality and psychological health in rehabilitation strategies.

However, a major limitation of our study is the relatively small sample size, which may limit the generalizability of the findings. Additionally, the neurological patient sample consisted exclusively of individuals with stroke, which does not allow for the results to be extended to all forms of neurological injury. Future studies may include a larger and more diverse sample to explore whether the observed trends are consistent in other populations with different neurological conditions.

## 5 Conclusions

In summary, this study investigated body representation and psychological distress between stroke patients and healthy subjects. The findings highlight the complexity of bodily and psychological experiences in health and disease contexts, suggesting the need for tailored clinical interventions to improve the quality of life of both groups. Future research may extend these findings by exploring the effectiveness of specific interventions to manage the identified issues.

## Author Contributions

**Maria Grazia Maggio**: conceptualization, investigation, writing–original draft, methodology, validation, visualization, writing–review and editing, software, formal analysis, project administration, data curation, supervision. **Amelia Rizzo**: conceptualization, investigation, writing–original draft, methodology, validation, visualization, writing–review and editing, software, formal analysis, project administration, data curation, supervision. **Morena De Francesco**: investigation, validation, visualization, writing–review and editing, data curation. **Martina Barbera**: investigation, validation, visualization, writing–review and editing. **Muhammad Kamran**: validation, visualization. **Rosaria De Luca**: validation, visualization. **Francesco Corallo**: validation, visualization. **Angelo Quartarone**: resources, supervision, validation, visualization, funding acquisition. **Rocco Salvatore Calabrò**: conceptualization, funding acquisition, writing–review and editing, visualization, validation, methodology, project administration, resources, supervision.

## Conflicts of Interest

The authors declare no conflicts of interest.

### Peer Review

The peer review history for this article is available at https://publons.com/publon/10.1002/brb3.70155.

## Data Availability

The data supporting the findings of this study are available on request from the corresponding author. The data are not publicly available due to privacy or ethical restrictions.
